# The effect of weight change on changes in breast density measures over menopause in a breast cancer screening cohort

**DOI:** 10.1186/s13058-015-0583-2

**Published:** 2015-05-30

**Authors:** Johanna Olga Pauline Wanders, Marije Fokje Bakker, Wouter Bernard Veldhuis, Petra Huberdina Maria Peeters, Carla Henrica van Gils

**Affiliations:** Julius Center for Health Sciences and Primary Care, University Medical Center Utrecht, Str. 6.131, P.O. Box 85500, 3508 GA Utrecht, The Netherlands; Department of Radiology, University Medical Center Utrecht, Room E01.132, P.O. Box 85500, 3508 GA Utrecht, The Netherlands; MRC-PHE Centre for Environment and Health, Department of Epidemiology and Biostatistics, School of Public Health, Imperial College London, St. Mary’s Campus, Norfolk Place, W2 1PG London, UK

## Abstract

**Introduction:**

High weight and high percentage mammographic breast density are both breast cancer risk factors but are negatively correlated. Therefore, we wanted to obtain more insight into this apparent paradox.

**Methods:**

We investigated in a longitudinal study how weight change over menopause is related to changes in mammographic breast features. Five hundred ninety-one participants of the EPIC-NL cohort were divided into three groups according to their prospectively measured weight change over menopause: (1) weight loss (more than −3.0 %), (2) stable weight (between −3.0 % and +3.0 %), and (3) weight gain (more than 3.0 %). SPSS GLM univariate analysis was used to determine both the mean breast measure changes in, and the trend over, the weight change groups.

**Results:**

Over a median period of 5 years, the mean changes in percent density in these groups were −5.0 % (95 % confidence interval (CI) −8.0; −2.1), −6.8 % (95 % CI −9.0; −4.5), and −10.2 % (95 % CI −12.5; −7.9), respectively (*P*-trend = 0.001). The mean changes in dense area were −16.7 cm^2^ (95 % CI −20.1; −13.4), −16.4 cm^2^ (95 % CI −18.9; −13.9), and −18.1 cm^2^ (95 % CI −20.6; −15.5), respectively (*P-trend* = 0.437). Finally, the mean changes in nondense area were −6.1 cm^2^ (95 % CI −11.9; −0.4), −0.6 cm^2^ (95 % CI −4.9; 3.8), and 5.3 cm^2^ (95 % CI 0.9; 9.8), respectively (*P-trend* < 0.001).

**Conclusions:**

Going through menopause is associated with a decrease in both percent density and dense area. Owing to an increase in the nondense tissue, the decrease in percent density is largest in women who gain weight. The decrease in dense area is not related to weight change. So the fact that both high percent density and high weight or weight gain are associated with high postmenopausal breast cancer risk can probably not be explained by an increase (or slower decrease) of dense area in women gaining weight compared with women losing weight or maintaining a stable weight. These results suggest that weight and dense area are presumably two independent postmenopausal breast cancer risk factors.

## Introduction

High mammographic breast density has been proven to be a strong independent risk factor for breast cancer [1, 2]. A woman’s breast consists of adipose, epithelial, and connective tissue. Adipose tissue is nondense tissue and appears translucent on a mammogram. Epithelial and connective tissue are radiodense and appear white on a mammogram. Percent density, which is determined by dividing the absolute dense area by the total breast area, multiplied by 100, is the most commonly used density measure. However, some argue that using dense area as a breast density measure is more appropriate since percent density is strongly influenced by the amount of adipose tissue in the breast. Both a higher absolute dense area and a higher percent density have been found to be associated with a higher breast cancer risk [3].

High weight, body mass index (BMI), and also adult weight gain are associated with a higher postmenopausal breast cancer risk [4–6]. At the same time, higher weight and BMI are associated with lower percent density since the measures of adiposity are associated with a higher breast fat content, resulting in a lower proportion of mammographic dense tissue [7–9]. So, although high weight and high mammographic percent density are both associated with higher postmenopausal breast cancer risk, they are inversely associated with each other [9–14].

Cross-sectional studies looking into the relation between BMI or weight and absolute dense area show mixed results. Some show that higher BMI or weight is significantly [8, 10, 12, 15–18] or nonsignificantly [9, 19] associated with a lower dense area, whereas others show that higher BMI or weight is associated with higher dense area [13, 20] or dense volume [16–19, 21, 22]. Longitudinal studies actually observing how breast measures change with a change in BMI or weight might give more insight into this process.

As far as we know, only one observational study (Reeves et al.) investigated the possible relation between natural (no organized intervention) changes in BMI and weight and changes in mammographic density measures [20]. They found that percent density decreases when BMI and weight increase in women transitioning through menopause and that there was no association between weight change and dense area.

An explanation for the apparent contradiction that high BMI and weight and weight gain, on the one hand, and high mammographic percent density, on the other hand, are associated with an increased postmenopausal breast cancer risk could be that dense area and the adiposity measures influence breast cancer risk independently from each other. Another explanation could be that increasing BMI and weight gain are accompanied by an increase, or slower decrease, in absolute dense area. Therefore, in this article, we investigated in a longitudinal design how changes in weight are related to changes in mammographic breast measures.

For this study, we used longitudinal data of women participating in the Prospect-EPIC study [23], which is part of the EPIC-NL prospective cohort study [24]. We selected all women who went through menopause within 5 years after recruitment. We focus on these women as large decreases in breast density and changes in weight are often observed around menopause [25–27]. The aim of this study is to investigate how percent density, absolute dense area, and absolute nondense area change over menopause and whether these changes differ between women who lose weight, maintain a stable weight, or gain weight over menopause.

## Methods

### Study population

The study population consisted of women participating in the Prospect-EPIC study [23], which is part of the EPIC-NL study [24]. EPIC-NL is the Dutch contribution to the European Prospective Investigation into Cancer and Nutrition (EPIC) study [28, 29]. The Prospect-EPIC cohort consists of 17,357 women recruited between 1993 and 1997 among breast cancer screening participants. In the Dutch national breast cancer screening program, women between 50 and 75 years of age are invited for a mammographic examination every 2 years. Prospect-EPIC participants lived in Utrecht (The Netherlands) or its vicinity and were between 49 and 70 years old at the time of recruitment. At the time of study enrolment, they were asked to complete a questionnaire containing questions about demographic, reproductive, and lifestyle factors and past and current morbidity. In addition, anthropometric parameters were measured by trained research nurses. About 5 years after recruitment, participants were asked to complete a similar questionnaire. This time, anthropometric measures were self-reported. All participants signed an informed consent form, and the study was approved by the Institutional Review Board of the University Medical Center Utrecht.

### Selection of study subjects

For this study, we used a study population that was described earlier [30, 31]. In brief, from the Prospect-EPIC cohort, women were selected who were pre- or peri-menopausal at the time of recruitment (being pre- or peri-menopausal was defined as having had at least one menses in the 12 months before recruitment) and who became postmenopausal after recruitment but before completing the first follow-up questionnaire approximately 5 years later. Women were considered postmenopausal when they did not have any menses during the 12 months prior to completing the follow-up questionnaire. The mammograms made at the time of recruitment were regarded as premenopausal mammograms, and the first mammogram after filling out the follow-up questionnaire was regarded as postmenopausal mammogram for all women. Women were excluded if they were current users of oral contraceptives or postmenopausal hormone therapy or used either less than 2 years before recruitment. Also, women having had bilateral ovariectomy were excluded, as were women with silicone prostheses or breasts that were too large to fit on a single mammogram. This resulted in 657 study participants [31]. In addition, we excluded women who stated in the baseline or follow-up questionnaire that they had ever used hormones for menopausal complaints (*n* = 57) and women who did not have pre- and post-menopausal weight information available (*n* = 9). This resulted in 591 study participants.

### Mammographic density readings

Mammographic density was determined as previously described [30]. Film screen mammograms were digitized with a laser film scanner (Lumiscan 50, Lumisys, Eastman Kodak Co., Rochester, NY, USA). We used the mediolateral oblique view of the left breast. Mediolateral oblique was, at that time, the routine view for breast cancer screening in The Netherlands. The sizes of the mammographic dense area and the total area of the breast were determined by using a computer-assisted threshold method [32]. Percent density was determined by dividing the size of the dense area by that of the total breast area, multiplied by 100. All mammograms, which were read in sets of 36 images in a random order, were assessed by one observer. Pre- and post-menopausal images of one woman were always in the same set [33]. Reliability of the reader was assessed by using a library set, which consisted of 36 randomly chosen films from the study subjects. The reader read this library set before the first set and five times between reading the other sets. The images in the library set were randomly ordered every time they were read, to prevent the observer from recognizing this set. In this study, the reader reached average intraclass correlation coefficients of 0.99 (range of 0.99–1.00) for total breast area, 0.81 (range of 0.75–0.86) for absolute dense area, and 0.90 (range of 0.88–0.92) for percentage breast density [30].

### Statistical analysis

First, descriptive statistics were performed. Then changes in breast measures over menopause were calculated by subtracting the premenopausal from the postmenopausal measure. Percentage weight change was determined by dividing the difference between post- and pre-menopausal weight by weight at baseline (premenopausal weight), multiplied by 100. Women were divided in ‘weight loss’ (women who lost more than 3 % of their baseline weight over menopause), ‘stable weight’ (women who lost or gained 3 % or less of their baseline weight), and ‘weight gain’ (women who gained more than 3 % of their baseline weight) groups. Previous studies used a change in weight of 2 kg as a reference group or stable weight group [34–37]. Since 2 kg is about 3 % of a body weight of 65 to 70 kg and the median baseline weight of our study population is 67 kg, we used 3 % weight change as a cutoff value. Means and 95 % confidence intervals (CIs) of changes in breast measures (percent breast density, dense breast area, and nondense breast area) were estimated for each weight change group. Means were determined and corrected for the premenopausal breast measure of interest and potential confounding factors. Age, BMI, and waist circumference at baseline, age at menarche, parity, and the number of years between pre- and post-menopausal mammogram were added to the model as continuous variables. Age at first childbirth was added to the model as a categorical variable (nulliparous, before age 21, between ages 21 and 25, between ages 26 and 30, between ages 31 and 35, and older than 35 years old). Physical activity was not added to the model, since it might be the cause of weight loss in women. Including it in the model could result in overadjustment.

In addition, we tested for linear trends in changes in breast measures over the weight change groups. SPSS GLM univariate analysis was used for determining both the mean changes in, and the trend over, the weight change groups.

Three sensitivity analyses were performed. The first was performed by dividing women in three weight change groups on the basis of their absolute weight change instead of their relative weight change. Means and linear trends were determined by using 2 kg as a cutoff value. This cutoff value was chosen because previous studies used a change in weight of 2 kg as a reference group or stable weight group [34–37]. In the second sensitivity analysis, women were divided in three weight change groups on the basis of a 4 % relative weight change. This cutoff was chosen to create a bigger contrast between the weight change groups while making sure that the group sizes did not become too small. Finally, we also performed linear regression analyses with changes in percent density, dense area, and nondense area as continuous dependent variables and weight change as a continuous independent variable. These regression models were adjusted for the same potential confounding factors as the factors mentioned before. To determine whether weight change might have different effects on density change among women who start out with a normal weight versus women who are overweight or obese at intake, we did a stratified analysis by BMI at intake (BMI of 25 kg/m^2^ or lower versus BMI above 25 kg/m^2^).

## Results

All women (*n* = 591) in this study were pre- or peri-menopausal at baseline and post-menopausal at follow-up. Our study population was comparable to the total group of Prospect-EPIC participants who went through menopause after baseline but before the first follow-up questionnaire with regard to age and weight at baseline and weight change. The follow-up questionnaires were filled out at a median of 4 years after baseline (interquartile range (IQR) of 3–5 years). The postmenopausal mammogram was taken at a median of 11 months after the follow-up questionnaire was filled out (IQR of 7–17 months). The median time between the pre- and post-menopausal mammogram was 5 years (IQR of 4–6 years).

Characteristics of the study population are presented per weight change group in Table 1. One hundred nine women lost more than 3 % of their baseline weight, 255 women maintained a stable weight (did not lose or gain more than 3 % of their baseline weight), and 227 women gained more than 3 % of their baseline weight over menopause. Age at baseline was comparable between weight change groups, as were age at menarche, number of children, age at first delivery, and time span between pre- and post-menopausal mammograms and baseline and follow-up questionnaires.

**Table 1:** Study population characteristics according to weight change categories (*n* = 591)

		Weight loss (more than −3.0 %)	Stable weight (−3.0 %; +3.0 %)	Weight gain (more than +3.0 %)
Number of women, n (%)		109 (18.5)	255 (43.1)	227 (38.4)
Weight change (%), median (IQR)		−5.8 (−8.1; −4.0)	0.0 (−1.5; 1.5)	6.3 (4.5; 10.3)
Weight change (kg), median (IQR)		−4.0 (−6.5; −3.0)	0.0 (−1.0; 1.0)	4.0 (3.0; 6.5)
Weight at baseline (kg), median (IQR)		69.0 (62.5; 82.0)	67.0 (61.5; 73.0)	66.0 (60.0; 71.5)
Age at baseline (years), median (IQR)		51 (50; 53)	51 (50; 53)	50 (50; 52)
BMI at baseline (kg/m^2^), median (IQR)		26.0 (23.0; 30.0)	25.0 (22.0; 27.0)	24.0 (23.0; 26.0)
BMI at follow-up^a^ (kg/m^2^), median (IQR)		24.3 (21.8; 27.6)	24.7 (22.3; 26.7)	25.9 (24.2; 28.4)
Waist at baseline (cm), median (IQR)		82.0 (75.0; 92.5)	80.0 (74.0; 86.0)	79.0 (73.0; 84.0)
Age at menarche (years), median (IQR)		13 (13; 14)	13 (12; 14)	13 (13; 14)
Age at menopause (years), median (IQR)		52 (50; 54)	52 (50; 54)	52 (50; 53)
Number of children		2 (2; 3)	2 (2; 3)	2 (2; 3)
Age at first delivery^b ^( years), median (IQR)		25 (23; 27)	25 (23; 27)	24 (22; 27)
Time span between pre- and post-menopausal mammogram (years), median (IQR)		5 (4; 6)	5 (4; 6)	5 (4; 6)
Time span between baseline and follow-up questionnaire (years), median (IQR)		4 (3; 5)	4 (3; 4)	4 (3; 5)
Age at first childbirth^b^, n (%)	No children	14 (12.8)	28 (11.0)	30 (13.2)
	≤20 years	8 (7.3)	14 (5.5)	23 (10.1)
	21–25 years	50 (49.5)	112 (43.9)	97 (42.7)
	26–30 years	30 (27.5)	79 (31.0)	61 (26.9)
	31–35 years	7 (6.4)	18 (7.1)	12 (5.3)
	>35 years	0 (0.0)	4 (1.6)	4 (1.8)

*IQR* interquartile range, *BMI* body mass index

^a^Calculated using body weight at follow-up and height at baseline

^b^Only among parous women

Pre- and post-menopausal breast and weight measures and the change in breast and weight measures are presented in Table 2. The median increase in weight is 1.0 kg (IQR of −1.0; 3.5) over menopause. Median percent density (−6.8 %, IQR of −15.6; 0.5), dense area (−12.4 cm^2^, IQR of −25.4; −4.1), and breast area (−9.6 cm^2^, IQR of −26.1; 2.1) all decreased over menopause, whereas the median amount of adipose (nondense) tissue slightly increased over menopause (0.3 cm^2^, IQR of −13.2; 14.8). Changes in breast measures over menopause per weight change group are presented in Table 3. The percent density decreased in all three weight change groups; the decrease was largest in the weight gain group and smallest in the weight loss group (*P*-trend = 0.001). The dense area decreased similarly among women in all weight change groups (*P*-trend = 0.437). There was an average decrease in nondense area in the weight loss group and to a smaller extent in the stable weight group as well, while the nondense area increased in the weight gain group. The change in nondense area is linearly associated with change in weight (*P*-trend < 0.001).

**Table 2:** Pre- and post-menopausal breast measures and changes in breast measures over menopause (*n* = 591)

	Median (IQR)		
	Premenopause at baseline	Postmenopause at follow-up	Median change over menopause^a^
Breast density, %	44.6 (27.6; 59.1)	33.6 (16.5; 52.4)	−6.8 (−15.6; 0.5)
Dense area, cm^2^	42.7 (30.4; 57.5)	26.8 (14.5; 37.3)	−12.4 (−25.1; −4.1)
Nondense area, cm^2^	57.3 (36.0; 88.5)	65.4 (34.0; 94.2)	0.3 (−13.2; 14.8)
Breast area, cm^2^	105.3 (84.2; 133.7)	94.6 (68.0; 120.1)	−9.6 (−26.1; 2.1)
Weight^b^, kg	67.0 (61.0; 73.0)	68.0 (62.0; 75.0)	1.0 (−1.0; 3.5)

*IQR* interquartile range

^a^Median change over menopause is determined by subtracting the pre- from the post-menopausal measures for each woman and then determining the median

^b^Information about postmenopausal weight is acquired from the follow-up questionnaire (self-reported). Weight change is the change in weight between the weight reported in the follow-up questionnaire and at baseline

**Table 3:** Changes in density measures over menopause in weight change groups (*n* = 591)

	Weight loss, more than −3.0 %	Stable weight, −3.0 %; +3.0 %	Weight gain, more than +3.0 %	P-trend
Number of women, n (%)	109 (18.5)	255 (43.1)	227 (38.4)	
Δ Breast density (%), mean (95 % CI)	−5.0 (−8.0; −2.1)	−6.8 (−9.0; −4.5)	−10.2 (−12.5; −7.9)	0.001
Δ Dense area (cm^2^), mean (95 % CI)	−16.7 (−20.1; −13.4)	−16.4 (−18.9; −13.9)	−18.1 (−20.6; −15.5)	0.437
Δ Nondense area (cm^2^), mean (95 % CI)	−6.1 (−11.9; −0.4)	−0.6 (−4.9; 3.8)	5.3 (0.9; 9.8)	<0.001

Means and 95 % confidence intervals (*CIs*) were adjusted for age, body mass index, and waist circumference at baseline, parity, age at first childbirth, time between pre- and post-menopausal mammogram, age at menarche, and the pre-menopausal breast measure of interest (premenopausal percentage breast density, dense area, or nondense area)

Similar results were found for weight change groups of 2 kg and 4 % change of weight (data not shown).

The results of the linear regression analyses with weight as a continuous independent variable were in line with the other results, showing that an increase in weight is statistically significantly associated with a decrease in percent density and an increase in nondense area. No association was found between weight change and change in dense area (Table 4).

**Table 4:** Association between percentage weight change and changes in breast (density) measures (*n* = 591)

	Regression coefficient	Standard error	95 % CI	*P* value
Δ Breast density, %				
Percentage weight change	−0.13	0.06	−0.25; −0.02	0.023
Δ Dense area, cm^2^				
Percentage weight change	−0.05	0.06	−0.18; 0.08	0.436
Δ Nondense area, cm^2^				
Percentage weight change	0.44	0.11	0.22; 0.65	<0.001

Regression coefficients and 95 % confidence intervals (*CIs*) were adjusted for age, body mass index, and waist circumference at baseline, parity, age at first childbirth, time between pre- and post-menopausal mammogram, age at menarche, and the premenopausal breast measure of interest (premenopausal percentage breast density, dense area, or nondense area)

The stratified analysis by BMI at intake did not lead to different conclusions when compared with the overall results shown in Table 3. The decrease in percent density is largest in women who gain weight and smallest in women who lose weight. In the two BMI groups, *P* values for change in percent density over the three weight change groups are 0.013 and 0.053, respectively. As the size of the change estimates in the two BMI groups are comparable (for BMI of not more than 25: −4.5 % (95 % CI −8.7; −0.3), −6.8 % (95 % CI −9.7; −3.8), and −9.9 % (95 % CI −12.7; −7.0) and for BMI of more than 25: −5.9 % (95 % CI −10.2; −1.6), −6.1 % (95 % CI −9.6; −2.6), and −10.4 % (95 % CI −12.8; −7.0) for the weight loss, stable weight, and weight gain groups, respectively), the borderline significance in the group with a BMI of more than 25 is probably caused by a lack of power because of the low number of women in this group. In both BMI groups, no linear trend was observed for change in dense area over the weight change groups. A statistically significant linear trend was observed in both BMI groups for the change in nondense area over the weight change groups (*P*_BMI ≤ 25_ = 0.006 and *P*_BMI > 25_ = 0.028). In both BMI groups, nondense area decreased in women who lost weight and increased in women who gained weight.

## Discussion

In this analysis of women going through menopause, both percent density and dense area decreased over menopause in all three weight change groups (loss, stable, and gain). An increase in weight was found to be associated with an increase in nondense (adipose) tissue and a decrease in percent density. No association was found between changes in weight and changes in dense area. Therefore, the larger decrease in percent density in women who gained weight than in women who remained stable or lost weight can probably be explained by changes in nondense area and not by a decrease in dense area.

A possible explanation for the apparent contradiction that both high weight and BMI and weight gain, on the one hand, and high mammographic percent density, on the other hand, are associated with a high postmenopausal breast cancer risk would be that weight gain is accompanied by an increase, or slower decrease, in absolute dense area. However, this possible explanation was not confirmed by our study results.

Our findings confirm the idea described by Reeves et al. that increases in weight appear to result in the accumulation of fat in the breast rather than altering the dense breast tissue [20]. To the best of our knowledge, only Reeves et al. investigated the association between changes in weight and breast density measures in a longitudinal observational design. They used data of 834 pre- and peri-menopausal women who were an average of 46.5 years old at enrollment and who were followed for an average of 4.8 years (standard deviation (SD) of 1.8). At the end of follow-up, 68.0 % of the women were late perimenopausal or postmenopausal. Annual change in weight was 0.32 kg (SD of 1.46), and annual changes in percent density and dense area were −1.14 % (SD of 3.60) and −0.77 cm^2^ (SD of 4.49), respectively. In our study population, the annual median changes in weight, percent density, and dense area were 0.20 kg, −1.36 %, and −2.48 cm^2^, respectively. The larger changes in percent density and, especially, dense area in our study population might be caused by our older study population (median age at baseline of 51 years and IQR of 50–53) and the fact that all women in our study population went through menopause compared with only 68.0 % in the study population of Reeves et al. Despite differences between the study populations, their results are comparable to ours. Reeves et al. observed also a negative association between changes in weight and changes in percentage breast density in women going through menopause and no association between changes in weight and changes in the absolute dense area. They did not study the association between weight and nondense area.

Two other studies on the associations between weight or BMI and changes in breast measures used trial data [7, 38]. Boyd et al. investigated the effect of a 2-year, low-fat, high-carbohydrate diet on breast density in 30- to 65-year-old women showing radiologic densities in at least 50 % of the breast area [38]. Woolcott et al. used data from a trial of a 12-month aerobic exercise intervention among postmenopausal women and looked at changes in BMI in relation to changes in both area and volumetric breast measures [7]. The time spans over which changes were measured were an average of 2.3 years (SD of 0.4) in the study by Boyd et al. and 1 year in the study by Woolcott et al. The intervention groups in both studies showed weight losses over the study period of −0.3 kg (no measure of statistical dispersion reported) and −2.3 kg (95 % CI −2.9; −1.7), respectively. The women in the control arm showed a weight gain (0.9 kg, no measure of statistical dispersion reported) and no significant change (−0.5 kg, 95 % CI −1.0; 0.1), respectively [38, 39]. In the study by Boyd et al., dense area changed by −3.74 cm^2^ (95 % CI −5.14; −2.35) in the intervention group and −1.27 cm^2^ (95 % CI −2.5; −0.1) in the control group during the study period. In a study by Woolcott et al., dense area changed by −0.3 cm^2^ (standard error of 0.7) in the intervention group and did not change in the control group (0.0 cm^2^, standard error of 0.7) [40]. Both studies found that a decrease in weight or BMI was associated with an increase in percent density and this is in agreement with our results. Regarding nondense area, Woolcott et al. also found comparable results; namely, a decrease in BMI was correlated with a reduction in nondense area. However, the association between weight or BMI and dense area showed contrary results: Woolcott et al. found that a decrease in BMI was correlated with an increase in dense area, whereas Boyd et al. found that weight loss was associated with a decrease in dense area, and we did not find an association between changes in weight and dense area at all.

An important difference between the study by Boyd et al. and our study is that in the former both the decrease in weight and the decrease in dense area are at least partly induced by the intervention, a low-fat high-carbohydrate diet. They do not show separate results for intervention and control groups in regard to the association between changes in weight and dense area. Therefore, it is unclear whether the significant relationship between weight change and change in dense area is confined to the intervention group or is observed in the control arm as well.

Woolcott et al. included only postmenopausal women, whereas our population goes through menopause during follow-up. Any dense area change caused by involution of glandular tissue during menopause might be much larger compared with changes due to weight change. We found a median decrease in dense area of 12.4 cm^2^ in 5 years (IQR of −25.1; −4.1), whereas Woolcott et al. found a change of only −0.1 cm^2^ (IQR of −4.1; 3.2) over 1 year. The estimated change over 5 years would be around −0.5 cm^2^. This might explain why we cannot detect an association between weight change and change in dense area in women going through menopause. However, Woolcott et al. found no significant correlation between change in BMI and change in dense volume, indicating that the negative relationship between change in BMI and dense area may also have been caused by chance.

Both high weight and dense area are postmenopausal breast cancer risk factors. Although less is known about the relationship between weight change and breast cancer risk, a recent meta-analysis shows evidence that weight gain too is significantly associated with higher postmenopausal breast cancer risk [5]. To investigate whether this relationship between high weight and weight gain, on the one hand, and breast cancer risk, on the other hand, might be mediated by dense area, we studied whether change in weight is associated with change in dense area. Our results show no association between changes in weight and changes in dense area. Therefore, it is unlikely that the effect of weight (gain) on breast cancer risk can be explained by (changes in) dense area. Weight (gain) and dense area are presumably two independent postmenopausal breast cancer risk factors.

We observed a strong relationship between weight change and a change in nondense area. For the last few years, the association between nondense area and breast cancer risk has been gaining attention in the literature. In 2014, authors of a published meta-analysis found that a larger nondense area could be inversely associated with breast cancer risk. However, it is still unclear whether this association is independent of the effect of the absolute dense area, since in most studies showing a protecting effect of nondense area on breast cancer risk, this effect disappeared after adjustment for dense area [3].

A strength of this study is its longitudinal design, enabling us to study prospectively the influence of weight change on changes in breast measures, and this is in contrast to cross-sectional studies. Two other strengths are that the sample size was almost 600 women and that all mammograms were read by the same observer.

A disadvantage of this study is that the breast measures may have been subject to measurement error since they were taken on digitized film-screen mammograms (FSMs). No information was available on the amount of radiation and compression force used during mammography. Since the amount of radiation and compression force can influence the breast density measurements, measurement errors might have occurred, causing misclassification. It is therefore important to confirm the results of this study by using volumetric breast measures from full-field digital mammograms. Another limitation is that postmenopausal mammograms were not taken on the same day as the reporting of weight. The first mammogram taken after the self-reported weight served as the postmenopausal mammogram. The median durations between weight reporting and postmenopausal mammogram were 11 months (IQR_stable_ 8; 17, IQR_gain_ 6; 17) in the stable weight and weight gain groups and 12 months (IQR_loss_ 6; 18) in the weight loss group. This might have led to random misclassification, attenuating the relationships under study. In addition, premenopausal weight was measured by a research nurse, and postmenopausal weight was self-reported. It is known that especially heavy women often underreport their weight. However, we did not find different associations between weight change and change in breast measures for women who start out with a normal weight and women who are overweight or obese at intake. Therefore, we think that in our study the influence of the limitation of self-reported weight on the conclusions is minimal.

## Conclusions

Going through menopause is associated with a decrease in both percent density and dense area. The decrease in percent density is largest in women who gain weight, due to an increase in the nondense tissue. The decrease in dense area is not related to weight change. So the fact that both high percent density and high weight or weight gain are associated with high postmenopausal breast cancer risk can probably not be explained by an increase (or slower decrease) of dense area in women gaining weight compared with women losing weight or maintaining a stable weight. These results suggest that weight and dense area are presumably two independent postmenopausal breast cancer risk factors. Whether an increase in nondense area, or breast fat tissue, has a role in breast cancer risk separately from general high weight or weight gain deserves further investigation.

### Ethics, consent, and permissions

All participants signed an informed consent form, and the study was approved by the Institutional Review Board of the University Medical Center Utrecht (protocol reference #93/090).

## Abbreviations

BMI: Body mass index
CI: Confidence interval
EPIC: European Prospective Investigation into Cancer and Nutrition
EPIC-NL: European Prospective Investigation into Cancer and Nutrition-The Netherlands
IQR: Interquartile range
SD: Standard deviation
